# Isolation and Genomic Characterization of Lytic *Caudoviricetes* Bacteriophage vB_Pae_YuaWU01 Targeting Multidrug-Resistant *Pseudomonas aeruginosa* from Hospital Sewage in Southern Thailand

**DOI:** 10.3390/life16050734

**Published:** 2026-04-28

**Authors:** Dechawat Wongprot, Benyapa Prakit, Morteza Saki, Chonticha Romyasamit

**Affiliations:** 1 College of Graduate Studies, Walailak University, Nakhon Si Thammarat 80160, Thailand; dechawat.wo@mail.wu.ac.th (D.W.); benyapa.pr@mail.wu.ac.th (B.P.); 2 Department of Microbiology, Faculty of Medicine, Ahvaz Jundishapur University of Medical Sciences, Ahvaz, Iran; mortezasaki1981@gmail.com; 3 Department of Medical Technology, School of Allied Health Sciences, Walailak University, Nakhon Si Thammarat 80160, Thailand; 4 Research Center in Tropical Pathobiology, Walailak University, Thasala District, Nakhon Si Thammarat 80160, Thailand

**Keywords:** bacteriophage, multidrug resistance, biofilm, *Pseudomonas aeruginosa*, whole genome sequencing

## Abstract

Globally, the rise in MDR *P. aeruginosa* infections poses a serious threat to public health, as these strains frequently exhibit extensive resistance to conventional antibiotics, prompting interest in bacteriophages as alternative treatments. In this study, we isolated and characterized a lytic *P. aeruginosa* phage, vB_Pae_YuaWU01, from hospital sewage in southern Thailand. Morphological analysis revealed *Siphovirus*-like characteristics. The phage demonstrated efficient host adsorption, with approximately 85.9% of particles attached within 15 min, and exhibited a latent period of 50 min with a burst size of 17.2 PFU/cell. It showed strong lytic activity, consistently suppressing bacterial growth without no regrowth observed over 72 h. Notably, the phage significantly inhibited biofilm formation by up to 59.9% and reduced pre-established biofilms by 39.78% at the highest tested concentration (10^9^ PFU/mL). Genome analysis revealed a 61,824 bp double-stranded DNA genome with 64.48% GC content and 88 predicted genes. Bioinformatic analysis suggests that the genome is organized into structural, replication, and lysis modules. Importantly, no toxin, antimicrobial resistance, lysogeny, or tRNA genes were identified, suggesting a favorable safety profile. The phage was classified within the genus *Yuavirus*, showing 97.4% genomic similarity to *Sphaerotilus* phage SN1, which infects a different host strain. The findings highlight its potential as a genetically safe therapeutic agent; however, its limited host range indicates that it may be best positioned as a strategic component of phage cocktails or as a synergistic partner with antibiotics to maximize therapeutic efficacy.

## 1. Introduction

Multidrug-resistant (MDR) pathogens become increasingly prevalent in both clinical and agricultural settings. The global health community faces the daunting prospect of a post-antibiotic age characterized by the loss of effective antimicrobial therapies, prompting the World Health Organization (WHO) to designate MDR *P. aeruginosa* as a high-priority antimicrobial-resistant pathogen. Carbapenem-resistant *P. aeruginosa* is classified as a high-priority pathogen due to its substantial mortality burden [1,2]. *P. aeruginosa* is a Gram-negative, aerobic, non–spore-forming rod that can cause a wide range of infections in both immunocompetent and immunocompromised hosts [3]. MDR *P. aeruginosa* is considered one of the most clinically significant bacterial pathogens, associated with high mortality rates due to its resistance to multiple antibiotics. This bacterium induces extensive tissue damage through various virulence factors, and its ability to form biofilms contributes to chronic, persistent, and antibiotic-resistant infections [4]. *P. aeruginosa* has been reported to contribute to about 7.1–7.3% of all healthcare-associated infections [5]. Data from the International Network for Optimal Resistance Monitoring (INFORM) indicate that multidrug-resistant *Pseudomonas aeruginosa* comprises approximately 11.5–24.7% of all isolates [6]. While each mechanism is associated with a specific class of antibiotics, multiple mechanisms collectively mediate varying levels of resistance across different antibiotic classes. Resistance mechanisms identified included OprD porin loss and the upregulation of efflux pumps, alongside the production of AmpC, ESBLs, and MBL-type carbapenemases [7]. *P. aeruginosa* contributes to the global dissemination of various antimicrobial resistance genes (ARGs) primarily through the horizontal gene transfer of mobile genetic elements frequently carrying ARGs, including *sul1, qnrS, bla*_VIM_*, bla*_TEM_*, bla*_CTX_, and *bla*_AIM-1_, which are associated with carbapenem resistance. Additionally, *tetA, ampC*, and *bla*_VIM_ are commonly linked to imipenem resistance [8,9].

Biofilm formation is a key factor contributing to antibiotic resistance in *P. aeruginosa*. The biofilm matrix limits antibiotic penetration, and cells within biofilms show reduced metabolic activity and increased expression of resistance-related genes. In addition, the biofilm environment promotes horizontal gene transfer. Altogether, these factors result in chronic infections that are difficult to treat using standard therapies [10]. Alternative therapeutic strategies have become necessary. Several non-antibiotic approaches have been explored, including traditional medicines, phytochemicals, nanoparticles, probiotics, and bacteriophages [11].

Bacteriophages are viruses that infect and lyse bacterial cells. Their narrow host specificity allows them to target harmful bacteria without affecting normal flora. Additionally, bacteriophages are considered highly safe, even with long-term use [12,13]. Bacteriophages have several advantages over antibiotics in treating bacterial infections, including strain specificity, lack of severe side effects, and low development costs. The current review shows that phage therapy is no longer an underestimated tool in treating bacterial infections [14,15]. Additionally, phages can target biofilm-forming bacteria, a major virulence factor in pathogens such as *P. aeruginosa*. Certain phages encode polysaccharide depolymerases that degrade the biofilm matrix, facilitating deeper penetration and enhanced bacterial clearance [16]. Phages can also disrupt quorum sensing, thereby impairing bacterial communication and virulence regulation [17].

This study was designed to isolate and characterize a bacteriophage against MDR *P. aeruginosa*. Beyond morphological characterization, we evaluated its in vitro lytic efficacy, antibiofilm potential, and genomic profile to assess its therapeutic suitability.

## 2. Materials and Methods

### 2.1. Bacterial Strains and Growth Conditions

*P. aeruginosa* ATCC15692 was obtained from the laboratory stock. Thirty-six MDR *P. aeruginosa* clinical isolates obtained from sputum specimens were previously confirmed as MDR by the hospital microbiology laboratory through routine diagnostic procedures. MDR was defined as acquiring non-susceptibility to at least one agent in three or more antimicrobial categories, as previously described [18]. All strains were cultured on nutrient agar (NA; HiMedia Laboratories, Mumbai, India) plates and incubated at 37 °C for 24 h under aerobic conditions. Bacterial colonies were then inoculated into 5 mL of nutrient broth (NB; HiMedia Laboratories, Mumbai, India) and incubated overnight at 37 °C for subsequent experiments. For extended storage, bacterial isolates were maintained in sterile 20% (*v*/*v*) glycerol at −80 °C.

### 2.2. Antibiotic Susceptibility of P. aeruginosa

The susceptibilities of *P. aeruginosa* strain to Meropenem (MEM; 10 µg), Amikacin (AMK; 30 µg), Imipenem (IPM; 10 µg) and Ciprofloxacin (CIP; 5 µg) were tested using the disc diffusion technique, and the results were interpreted in accordance with the 2024 guidelines of the Clinical and Laboratory Standards Institute (CLSI) [19].

### 2.3. Bacteriophage Isolation, Purification, and Propagation

Hospital sewage samples were collected from a hospital in Nakhon Si Thammarat, southern Thailand. The samples were centrifuged at 4000× *g* at 4 °C for 30 min to remove debris. The supernatant was sterilized by filtration through a 0.22 μm pore size membrane filter (Sigma-Aldrich, Merck KGaA, Darmstadt, Germany). One mL of filtered sewage was mixed with 200 μL of *P. aeruginosa* and adsorbed for 15 min. A double-layer agar technique was employed, using a 5 mL soft agar overlay. Following incubation at 37 °C for 24 h, the presence of visible plaques was recorded as a positive result for phage isolation.

For purification, a single plaque was picked using a sterile pipette tip and suspended in 1 mL of sodium–magnesium (SM) buffer. After overnight incubation at 4 °C, samples were centrifuged (4000 rpm, 30 min, 4 °C), and the supernatant was filtered through a 0.22 μm syringe filter. Three rounds of purification were performed to obtain purified phage preparation.

To prepare high-titer phage stocks, ten-fold serial dilutions of the purified phage were performed in SM buffer. A 100 μL of the diluted phage was combined with 200 μL of bacterial culture in soft agar. The mixture was immediately overlaid onto an NA plate and incubated at 37 °C for 24 h. Phage particles were eluted by flooding the semi-confluent plates with 5 mL of SM buffer. The suspension was collected and centrifugation at 4000× *g* for 30 min at 4 °C to ensure the complete removal of bacterial debris. The supernatant was filtered through a sterile 0.22 μm pore-size membrane filter, collected as phage stock, and stored at 4 °C until use.

### 2.4. Bacteriophage Morphological Characterization

Phage morphology was examined by depositing a drop of the lysate onto carbon-coated copper grids, followed by negative staining with 2% (*w*/*v*) uranyl acetate (pH 6.7). The phage particles were visualized using a Talos F200i electron microscope (Thermo Fisher Scientific, Waltham, MA, USA) operating at a voltage of 200 kV.

### 2.5. Bacteriophage Host Range Determination

Host range of the phage was evaluated against 36 clinical isolates of MDR *P. aeruginosa* using the standard spot assay [20]. Briefly, 10 µL of phage lysate (>10^8^ PFU/mL) was spotted onto a bacterial lawn prepared with soft agar. After overnight incubation at 37 °C, the plates were examined for the presence of clear zones or plaques to determine the susceptibility of each bacterial strain.

### 2.6. Bacteriophage Adsorption Rate Assay

The adsorption assay followed a previously established protocol [21,22]. The phage was mixed with *P. aeruginosa* at an (MOI) of 0.01 and incubated at 37 °C. At each time point of incubation (0, 1, 2, 5, 7.5, 10, 15, 20, 25, and 30 min), the mixture was aliquoted and pelleted by centrifugation at 15,000× *g* for 2 min. The supernatant was serially diluted in the SM buffer, and plaque assay was performed to determine the number of free phage particles in the supernatant. The adsorption rate constant (*k*; mL/min) was computed using the formula reported in a previous study [23].

$$k=\frac{2.3\log Po}{BtP}$$

where *k* is the adsorption rate constant (mL/min); B is the bacterial cell concentration; *t* is the time; Po is the stating titer; P is the final titer.

### 2.7. One-Step Growth Curve

A one-step growth curve was performed to characterize the latent period, and average burst size was calculated following the previously described method [21,24]. *P. aeruginosa* was infected with the phage at an MOI of 0.1 for 10 min at 37 °C. Then, the cells were centrifuged at 12,000× *g* for 5 min. The pellet was resuspended in NB and incubated in a shaker at 150 rpm at 37 °C. Samples were collected every 10 min (including the 0 min time point) for a total of 120 min, and the phage titer was determined using the double-layer agar method. The burst size was calculated using the following equation.


$$\text{Burst size}=\frac{\text{Initial infected cell count}\;(\text{PFU}/\text{mL})}{\text{Final phage titer}\;(\text{PFU}/\text{mL})}$$

### 2.8. Killing Activity of the Bacteriophage

The impact of the phage on *P. aeruginosa* growth was evaluated using a method adapted from previous study [21]. *P. aeruginosa* cultures were cultured at 37 °C for 24 h, with the bacterial inoculum adjusted to a final concentration of 1 × 10^8^ CFU/mL. The bacteria were then exposed to phage at MOIs of 0.01, 0.1, 1, and 10. Bacterial growth was monitored hourly by measuring OD600 during 72 h of incubation.

### 2.9. Scanning Electron Microscopy (SEM)

To examine the lytic impact of the phage, the structure of *P. aeruginosa* was analyzed via scanning electron microscopy (SEM) according to the method described previously [21]. Phage-infected *P. aeruginosa* (MOI 1, 4 h, 37 °C) was recovered via centrifugation at 4000× *g* for 20 min. The bacterial pellets were washed twice with 1 mL of PBS and resuspended in 200 μL of the SM buffer. A suspension of bacteria was dispensed onto sterile glass slides and fixed with 2.5% (*v*/*v*) glutaraldehyde. Following fixation, the cells were rinsed twice with PBS. Subsequently, dehydrated using a graded ethanol series (20–100%, *v*/*v*). Critical point drying was employed for sample stabilization, after which a thin layer of gold was deposited via sputter coating. The morphology of the bacterial cells was observed using a field-emission scanning electron microscope (FE-SEM) (Merlin VP Compact, Carl Zeiss AG, Oberkochen, Germany).

### 2.10. Antibiofilm Activity of Phage vB_Pae_YuaWU01

The antibiofilm efficacy of the phage, specifically its ability to inhibit initial formation and eradicate mature biofilms, was assessed using the crystal violet (CV) method as previously described [24]. For the inhibition assay, a 1:1 volume ratio of bacterial suspension and phage was used. Specifically, 100 µL of *P. aeruginosa* (10^8^ CFU/mL) was co-incubated with 100 µL of phage suspension (10^5^–10^9^ PFU/mL) in 96-well plates at 37 °C for 24 h. For eradication, 24 h preformed biofilms were washed twice with PBS, treated with phage (10^5^–10^9^ PFU/mL), and incubated for another 24 h. Post treatment, biofilms were washed, air dried, and stained with 0.1% CV for 30 min. The bound dye was solubilized in absolute ethanol, and the biofilm biomass was quantified by measuring the optical density at 570 nm.

### 2.11. Phage Whole-Genome Sequencing

Phage genomic DNA was extracted using the DNeasy Blood & Tissue Kit (Qiagen, Hilden, Germany) according to the manufacturer’s instructions, with minor modifications [25]. DNA libraries were constructed using the TruSeq Nano DNA Library Prep Kit (Illumina, San Diego, CA, USA). After verifying the library quality, sequencing was performed, and the raw sequence data quality from high-throughput sequencing pipelines was assessed using FastQC (version 0.11.5). Genome Sequencing and Bioinformatic Analysis. The phage genome was assembled and screened for genetic profiles, including virulence factors and antimicrobial resistance (AMR) genes, using the Phagenomics pipeline (https://www.phagenomics.net; accessed on 10 November 2025). Genome annotation was performed using Pharokka v1.2 via the Phagenomic pipeline and Bakta v1.8 via the Galaxy platform (https://usegalaxy.eu/; accessed on 10 November 2025). Genome visualization was conducted using Proksee (https://proksee.ca; accessed on 3 December 2025), while putative functions of [26] hypothetical proteins were assigned via HHpred (https://toolkit.tuebingen.mpg.de/tools/hhpred; accessed on 26 November 2025). To determine evolutionary relationships, a phylogenomic tree was constructed using the VICTOR webserver (https://victor.dsmz.de; accessed on 25 November 2025), and genomic similarity with the closest relatives was evaluated using Average Nucleotide Identity (ANI) version 1.1.0 [27]. The safety profile, including the presence of virulence factors, drug resistance genes, and tRNA sequences, was rigorously analyzed using VirulenceFinder version 2.2.9 (https://www.mgc.ac.cn/VFs/main.htm; accessed on 16 December 2025), ResFinder version 4.7.2 (http://genepi.food.dtu.dk/resfinder; accessed on 16 December 2025), and tRNAscan-SE (https://lowelab.ucsc.edu/tRNAscan-SE/; accessed on 16 December 2025) to ensure the genetic safety of the phage. The phage genome sequence has been deposited in GenBank under accession number PZ284855.

### 2.12. Statistical Analyses

Statistical analyses were conducted using GraphPad Prism 10. Data are expressed as mean ± SEM from three independent biological replicates, each with three technical replicates (n = 3). Statistical significance was determined using one-way ANOVA followed by Dunnett’s post hoc test for comparisons between phage-treated groups and the untreated control, and two-way ANOVA followed by Dunnett’s post hoc test for experiments involving two independent variables (e.g., time and treatment). Significance notations are defined as a * *p* < 0.05.

## 3. Results

### 3.1. Antibiotic Susceptibility of P. aeruginosa ATCC15692

*P. aeruginosa* ATCC15692 was resistant to MEM (Carbapenem) indicating that *P. aeruginosa* ATCC15692 strain was a carbapenem-resistant *P. aeruginosa* (CRPA) strain. All clinical isolates included in this study were previously confirmed as MDR *P. aeruginosa* by the hospital microbiology laboratory.

### 3.2. Isolation of Bacteriophages

**Figure 1:**
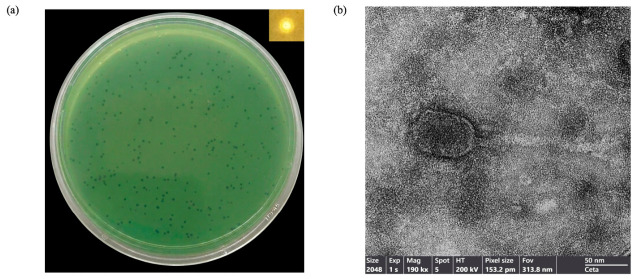
(**a**) Plaques of phage vB_Pae_YuaWU01 on *P. aeruginosa* lawn. (**b**) Transmission electron micrographs of vB_Pae_YuaWU01.

Phage vB_Pae_YuaWU01 was isolated from hospital sewage, and *P. aeruginosa* ATCC15692 was used as the host strain. Plaque assay revealed clear lytic zones with a consistent diameter of 1–2 mm with surrounding halo. The structural morphology of the bacteriophage was visualized using transmission electron microscopy. The phage has a *Siphoviridae*-like morphology, characterized by an icosahedral head with a diameter of approximately 89.93 ± 0.3 nm and a flexible, non-contractile tail with a length of approximately 120.45 ± 0.2 nm (Figure 1).

### 3.3. Phage Host Range Determination

The host range of Phage vB_Pae_YuaWU01 was assessed by spot test against 36 MDR *P. aeruginosa* clinical isolate strains (Table S1, Figure S1). The results revealed that the phage exhibited lytic activity against 22.22% of MDR *P. aeruginosa* strains, suggesting that the phage has potential for controlling MDR *P. aeruginosa* infections.

### 3.4. Phage Adsorption

**Figure 2:**
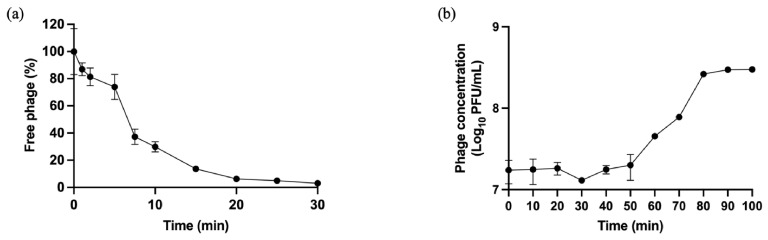
(**a**) Adsorption kinetics of phage vB_Pae_YuaWU01. (**b**) One-step growth curve of phage vB_Pae_YuaWU01 revealing the latent period and burst size.

The efficiency and rate of phage adsorption onto *P. aeruginosa* were evaluated. Approximately 70.4% of the phage particles adsorbed to the host cells within 10 min. Adsorption kinetics revealed that over 85.9% of phage particles had attached to the host cells within 15 min of incubation (Figure 2a). The calculated value of the adsorption rate constant (*k*) was 1.04 × 10^−9^ mL/min.

### 3.5. One-Step Growth Curve

To assess infection dynamics in the primary host strain, the one-step growth curve of phage vB_Pae_YuaWU01 was determined. The results showed an initial latent period of 50 min. This was followed by a rapid rise in phage progeny during the burst phase, reaching a peak titer of 2.99 × 10^8^ PFU/mL at 100 min. The calculated burst size was 17.2 phage particles per infected cells (Figure 2b).

### 3.6. Killing Activity of Phage vB_Pae_YuaWU01 Against P. aeruginosa

**Figure 3:**
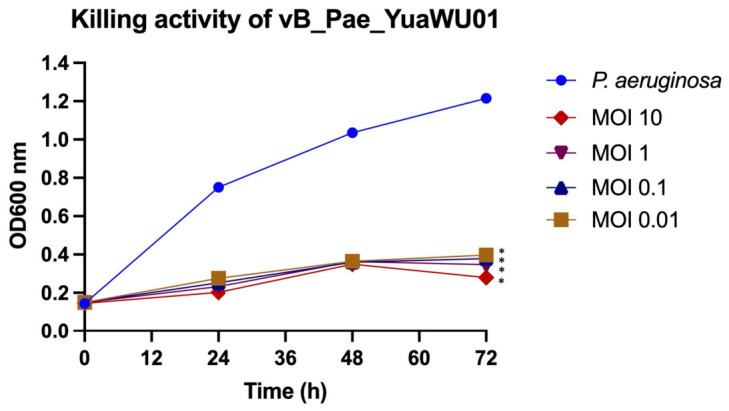
In vitro killing activity of bacteriophage vB_Pae_YuaWU01 against *P. aeruginosa* at different MOIs. The data show the mean ± SEM (* *p* value < 0.05).

The bacteriolytic activity of the phage was assessed through killing curve assays using *P. aeruginosa* at MOIs of 0.01, 0.1, 1, and 10 (Figure 3). In the absence of phage infection, the *P. aeruginosa* control exhibited continuous exponential growth, with OD600 values increasing steadily throughout the 72 h incubation, whereas phage-infected cultures exhibited dose-dependent growth inhibition. At each time point post-infection, all phage-treated groups showed a lower turbidity compared to the control, with the most significant suppression observed at MOIs of 1 and 10. Notably, at MOIs of 1 and 10, the turbidity at 72 h was even lower than at 48 h. Furthermore, no significant bacterial regrowth was observed even at the lowest MOI (0.01), suggesting that the phage effectively suppresses *P. aeruginosa* under these conditions.

### 3.7. Structural Changes in P. aeruginosa Cells Post Phage vB_Pae_YuaWU01 Infection

**Figure 4:**
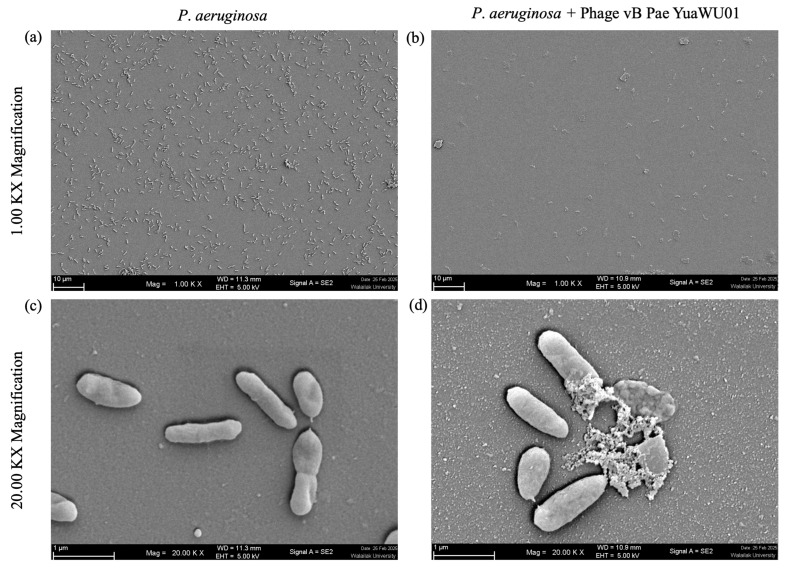
Morphology of *P. aeruginosa* cells under SEM. (**a**,**c**) Untreated *P. aeruginosa* cells. (**b**,**d**) *P. aeruginosa* infected with Phage vB_Pae_YuaWU01. The cells were observed at magnifications of ×1000 and ×20,000.

SEM analysis revealed that, at 1.00 KX magnification, bacterial titers were significantly reduced following phage infection compared to the untreated control group (Figure 4a,b). While at 20.00 KX magnification, untreated *P. aeruginosa* showed typical rod-shaped architecture with a smooth, undamaged exterior with a diameter of 0.291 ± 0.003 μm and a length of 1.257 ± 0.003 μm (n = 3) (Figure 4c), those infected with phage exhibited clear evidence of cell lysis. The phage-treated cells showed a morphological change (diameter 0.474 ± 0.010 μm and length 0.852 ± 0.010 μm (n = 3) distinct structural damage, including membrane blisters and pore formation, resulting from intracellular phage replication. These morphological changes confirm the potent lytic activity of phage vB_Pae_YuaWU01, ultimately triggering extensive cellular lysis and subsequent bacterial death (Figure 4d).

### 3.8. Antibiofilm Activity of Phage vB_Pae_YuaWU01

**Figure 5:**
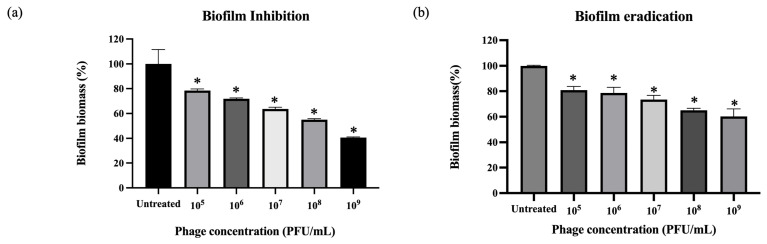
Evaluation of phage antibiofilm activity. Percentage of biofilm biomass remaining after treatment with different phage titers in (**a**) inhibition and (**b**) eradication models. The untreated group represents 100% biomass. Significant reductions were observed across all tested concentrations. The data show the mean ± SEM (* *p* value < 0.05).

The antibiofilm activity of the phage was evaluated through both inhibition and eradication assays. Results exhibited a significant, dose-dependent reduction in biofilm biomass across both models (Figure 5). Biofilm inhibition, the phage effectively prevented biofilm formation, reaching its highest inhibition at 10^9^ PFU/mL with only 40.1% biomass remaining. Even at the lowest concentration of 10^5^ PFU/mL, the phage still reduced the biofilm by 22.2%. Biofilm eradication, the phage also demonstrated a robust ability to disrupt mature, pre-established biofilms. At 10^9^ PFU/mL, the biofilm biomass was significantly reduced to 60.22%. Although mature biofilms were more difficult to remove, the phage still showed a clear effect at 10^5^ PFU/mL, achieving a 19.17% reduction.

### 3.9. Whole-Genome Sequencing and Analysis

**Figure 6:**
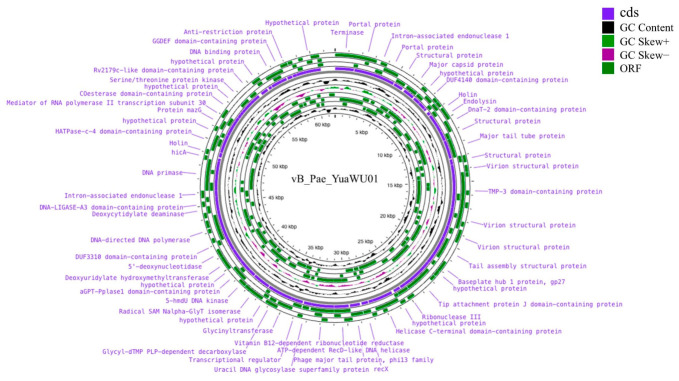
The circular genome map of phage vB_Pae_YuaWU01.

Genomic annotation was performed using Bakta and PHAROKA (Table S2, Figure 6), while putative functions of hypothetical proteins were further predicted using HHpred (Table S4). To maximize functional coverage, a three-tier annotation strategy was employed. Pharokka v1.2 and Bakta v1.8 were applied in parallel to capture both phage-specific and general gene functions, followed by HHpred-based remote homology detection for remaining hypothetical proteins. Pharokka alone left 41 of 88 ORFs (46.6%) uncharacterized; the addition of Bakta reduced this to 7 (8.0%); and HHpred subsequently resolved 2 additional ORFs at ≥95% probability, yielding a final hypothetical protein proportion of only 5.7% (5/88), such that 94.3% of ORFs were functionally assigned in total. Together, this analysis revealed a 61,824 bp double-stranded DNA genome with a GC content of 64.48% and 88 predicted open reading frames (ORFs) organized into distinct functional modules. The DNA packaging module includes the terminase large subunit (ORF 1) and portal protein (ORF 2), followed by the head module, which comprises the major head protein (ORF 7), scaffolding protein (ORF 6), head morphogenesis protein (ORF 5), and head-tail adaptors (ORFs 14 and 15). The tail module, the largest functional group, contains essential structural components such as the tail length tape measure protein (ORF 21), tail terminator (ORF 16), tail assembly proteins (ORFs 24 and 25), and various tail structural proteins (ORFs 17–20 and 28). Host cell lysis is governed by a lysis module consisting of a three-component system: spanin (ORF 11), holin (ORF 12), and endolysin (ORF 13). Furthermore, the DNA metabolism module facilitates genome replication and regulation; it features DNA helicase (ORF 32, 56), DNA primase (ORF 59), DNA polymerases (ORFs 53), and DNA repair photolyase (ORF 45), alongside DNA-modifying enzymes such as homing endonuclease (ORF 4) and HNH endonuclease (ORF 44). In addition, no toxin, drug-resistance, tRNA, or lysogeny-associated genes were identified in the phage vB_Pae_YuaWU01 genome.

### 3.10. Phylogenetic Tree

**Figure 7:**
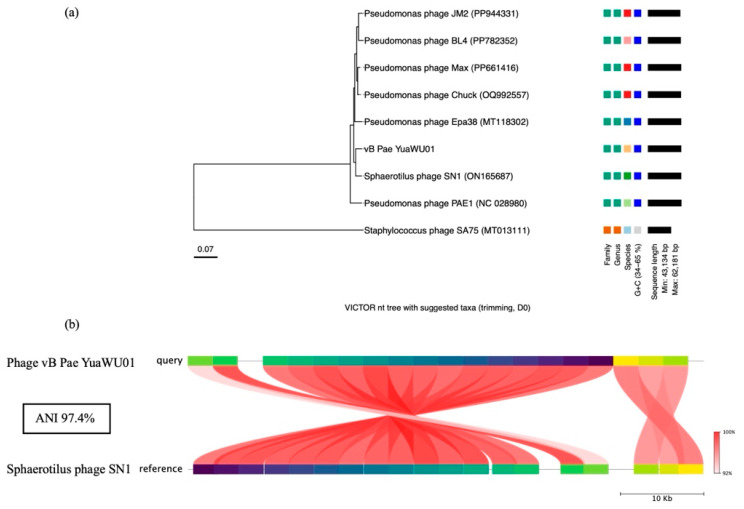
(**a**) Phylogenetic tree analysis of the whole-genome sequence of phage vB_Pae_YuaWU01 and related phages. (**b**) Average Nucleotide Identity (ANI) comparison of phage vB_Pae_YuaWU01 and its closely related phage. High ANI values indicate strong genetic similarity. Scale bars: 10 Kb.

To evaluate the genomic similarity between vB_Pae_YuaWU01 and related bacteriophages, the complete genome sequence was searched against the NCBI BLASTn database (version 2.17.0). Comprehensive phylogenomic analysis was subsequently performed using the VICTOR (Virus Classification and Tree Building Online Resource) server, a genomic pipeline for genome-based phylogeny and the classification of prokaryotic viruses. The phage was the most closely related to *Sphaerotilus* phage SN1 (ON165687) (Figure 7a). Other related phages to this phage were *Pseudomonas* phage PAE1 (NC 028980), *Pseudomonas* phage Epa38 (MT118302), *Pseudomonas* phage Chuck (OQ992557), *Pseudomonas* phage Max (PP661416), *Pseudomonas* phage BL4 (PP782352), and *Pseudomonas* phage JM2 (PP944331), respectively. *Staphylococcus* phage SA75 (MT013111) was employed as the outgroup. Genomic analysis identified this phage as a member of the genus *Yuavirus*, subfamily *Rabinowitzvirinae*, family *Mesyanzhinovviridae*, class *Caudoviricetes*, and order *Caudovirales*. The genome of this phage was compared to its closest relative, *Sphaerotilus* phage SN1, using FastANI. Both phages shared a high overall identity of 97.4%. Most genomic regions were nearly identical, although some specific areas showed lower similarity (Figure 7b).

## 4. Discussion

The increasing emergence of antimicrobial resistance has become a major global public health concern, particularly in healthcare-associated infection (HAIs). *P. aeruginosa* is a significant cause of morbidity and mortality in people with cystic fibrosis (CF) [28]. In cases of ventilator-associated pneumonia (VAP), infections caused by *P. aeruginosa* are associated with mortality rates of 34–48% [29]. Additionally, *P. aeruginosa* bacteremia has a mortality rate exceeding 33%, which can double when caused by MDR strains [30]. In response, research has explored alternatives or adjuvants to antibiotics, including bacteriophages. These highly bacteria-specific viruses have gained significant interest in recent years. In this study, we successfully isolated and characterized a virulent lytic phage against MDR *P. aeruginosa*, providing its biological, genomic, and antibacterial mechanisms of the phage.

To highlight the distinctiveness of vB_Pae_YuaWU01, we compared it with previously reported *P. aeruginosa* phages in Table 1

Phage vB_Pae_YuaWU01, which possesses an icosahedral head and a long noncontractile tail, was successfully isolated from hospital sewage in southern Thailand. Morphological analysis via transmission electron microscopy (TEM) confirmed its classification within the class *Caudoviricetes* [36].

The presence of a halo surrounding these plaques suggests that vB_Pae_YuaWU01 may encode tail-associated polysaccharide depolymerases capable of degrading extracellular polymeric substances (EPS) [37]. This enzymatic activity is a critical feature for penetrating the complex matrix of bacterial biofilms, thereby enhancing the phage’s therapeutic potential against persistent infections [38]. The phage showed a limited host range, similar to phage Lydia within the genus *Yuavirus* [31]. This narrow host range likely reflects a high degree of receptor specificity, in which the phage’s receptor-binding proteins (RBPs) recognize specific surface structures, such as Type IV pili or lipopolysaccharides, present only on certain MDR *P. aeruginosa* strains. Despite its restricted host range, the phage offers the benefit of high specificity, which ensures targeted lytic activity without disrupting the host’s commensal microbiota. However, it exhibited lytic activity against genetically diverse MDR *P. aeruginosa* clinical strains. Moreover, personalized phage therapy can be applied, and the phage may serve as a complementary component, especially as part of a phage cocktail to broaden host range and enhance efficacy against MDR *P. aeruginosa* strains [39].

In this study, phage vB_Pae_YuaWU01 demonstrated high adsorption efficiency, characterized by rapid binding to *P. aeruginosa*. This rapid attachment indicates a high affinity for specific surface receptors; this rapid attachment allows the phage to start the infection quickly, which may help it avoid being cleared by the host’s immune system [40]. The phage has long latent period and a moderate burst size. Although these parameters are lower than those reported for certain highly virulent *Pseudomonas* phages, the biological performance of the phage remained strongly lytic across all infection conditions. Notably, the phage exhibited sustained bacterial suppression for up to 72 h, even at low MOIs, with no bacterial regrowth observed. However, since bacterial growth was monitored by OD600 alone, the possible emergence of phage-resistant variants cannot be ruled out. Future studies using CFU and PFU measurements would be needed to confirm these findings. Moreover, phages can reduce the severity of infections even after bacterial resistance evolves. In some cases, phages can themselves evolve to overcome this resistance, thereby continuing to suppress bacterial populations [41,42]. Even when resistance occurs, it often results in fitness trade-offs that reduce bacterial growth rates and virulence such as alterations or loss of bacterial surface structures commonly associated with phage resistance can impair nutrient uptake, biofilm formation, and binding to host receptors. This can lead to the attenuation of bacterial virulence [43,44], increased hypersensitivity to antibiotics [45,46], which would be beneficial for subsequent treatments.

*P. aeruginosa* is known for its ability to form strong biofilms, which play a critical role in its persistence and resistance to antimicrobial treatments. The mechanism of biofilm formation provides a protective extracellular matrix that limits antimicrobial penetration and enhances bacterial survival, making infections difficult to eradicate [37]. In the present study, the results indicated that the phage effectively reduced and prevented biofilm formation, with a significant reduction in biofilm biomass observed at high phage concentrations, indicating that phages may disrupt biofilm formation by early attachment stage and reducing the population of planktonic cells required for biofilm maturation. The results of this study are consistent with previous reports demonstrating the effectiveness of phages against biofilm-forming *P. aeruginosa*. Similarly, phage phPA-Intesti has been reported to achieve substantial reductions in both planktonic cells and biofilms of *P. aeruginosa*, including up to 5.5 log CFU/cm^2^ in biofilm models. Collectively, these findings highlight the potential of bacteriophages as an alternative strategy for controlling biofilm-associated and MDR *P. aeruginosa* infections [47].

The whole genome of phage vB_Pae_YuaWU01 was studied, revealing that it belongs to the genus *Yuavirus*, subfamily *Rabinowitzvirinae*, family *Mesyanzhinovviridae*, class *Caudoviricetes*. Phylogenetic analysis classified the phage to the genus *Yuavirus*, a group typically associated with *P. aeruginosa*. However, comparative genomics revealed an unexpected and high degree of similarity to *Sphaerotilus* phage SN1 [48], which infects the Betaproteobacterium *Sphaerotilus natans*. This interspecific order genetic relationship underscores the prevalence of Horizontal Gene Transfer (HGT) and modular evolution within the class *Caudoviricetes*. Hospital sewage acts as an evolutionary “hotspot” that drives these genetic connections. Because sewage contains a high density of diverse bacteria and phages, it creates a perfect environment for them to swap functional modules, the genetic building blocks for replication and structure. Through this “mosaic” evolution, the phage can pick up beneficial traits from a shared environmental gene pool, making it more adaptable to changing conditions [49].

Genomic analysis revealed that vB_Pae_YuaWU01 carries well-organized modules associated with DNA packaging, head and tail morphogenesis, replication, and host lysis, reflecting conserved *Caudoviricetes* architecture. Among the lysis components, the endolysin stands out as functionally significant. Endolysins act by cleaving peptidoglycan to trigger host cell lysis, and phage-derived lysins have demonstrated potent antimicrobial activity against *P. aeruginosa* independent of whole-phage infection [50]. This highlights an additional therapeutic dimension: this phage may serve not only as a whole phage therapy candidate but also as a source of lysin-based antimicrobials, a strategy increasingly supported in the current literature. Moreover, its safe genetic profile, lacking antimicrobial resistance genes, virulence determinants, and tRNA sequences, further underscores its suitability and biosafety for therapeutic application.

## 5. Conclusions

Phage vB_Pae_YuaWU01 demonstrated a high capacity to suppress bacterial growth and disrupt biofilm formation in *P. aeruginosa*, while possessing a favorable safety profile, highlighting its potential as a therapeutic candidate. It is detailed genomic characterization and absence of undesirable genetic elements. However, its narrow host range indicates that it may be more suitable as part of a phage cocktail or in synergy with antibiotics.

## Figures and Tables

**Table 1:** Comprehensive comparison of vB_Pae_YuaWU01 with previously reported *P. aeruginosa* phages.

Phage	Geographic Origin	Isolation Source	Phage Type	Genus	Morphology	Closest Relative	Annotation Platform(s)	Annotated ORFs (%)	Biofilm	References
Phage Lydia	Russia	Selenga River	Lytic	*Yuavirus*	*Siphovirus*	*Pseudomonas virus* M6	BLAST + HHpred	62.9% (56/89)	No	[31]
EPa38	USA	Lake water	Lytic (likely)	*Yuavirus*	*Siphovirus*	N/R	N/R	N/R	N/R	[32]
NEU2024	Nicosia, Cyprus	Hospital wastewater	Lytic	*Yuavirus*	*Siphovirus*	*Yuavirus* *members*	BLASTp	48.9% (45/92)	No	[33]
Phage_Pae01	China	Hospital sewage	Lytic	*Pbunavirus*	*Myovirus*	*Pseudomonas phages* vB_PaeM_C2-10_Ab02	BLASTp	32.4% (57/176)	Yes	[34]
Baskent_P4_1	Turkey	Wastewater	Lytic	*Angoravirus*	*Siphovirus*	*Stenotrophomonas* phage	BLASTp	N/R (53/N/A)	Yes	[35]
vB_Pae_YuaWU01 [THIS STUDY]	Southern Thailand	Hospital sewage	Lytic	*Yuavirus*	*Siphovirus*	*Sphaerotilus* phage SN1	Bakta + Pharokka + HHpred	94.3% (83/88)	Yes	This study

N/A = Not applicable. N/R = Not reported.

## Data Availability

Data included in article/Supplementary Material/referenced in article.

## Supplementary Materials

The following supporting information can be downloaded at: https://www.mdpi.com/article/10.3390/life16050734/s1, Figure S1. Host range of phage vB_Pae_YuaWU01; Table S1: Host range of phage vB_Pae_YuaWU01; Table S2. Comparison of phage gene annotations produced by Bakta and Pharokka; Table S3: Nucleotide and amino acid sequences of predicted open reading frames (ORFs) of phage vB_Pae_YuaWU01; Table S4: HHpred analysis results for hypothetical proteins.
