# Supplementary material for: Isolation and Genomic Characterization of Lytic Caudoviricetes Bacteriophage vB_Pae_YuaWU01 Targeting Multidrug-Resistant Pseudomonas aeruginosa from Hospital Sewage in Southern Thailand
Source: Life (Basel). 2026 Apr 28;16(5):734. doi: 10.3390/life16050734 (PMC13208608; doi:10.3390/life16050734)
Supplement: Supplementary file 1 [file life-16-00734-s001.zip › Supplementary figure.pdf]

Supplementary Figure S1. Host range of phage vB_Pae_YuaWU01

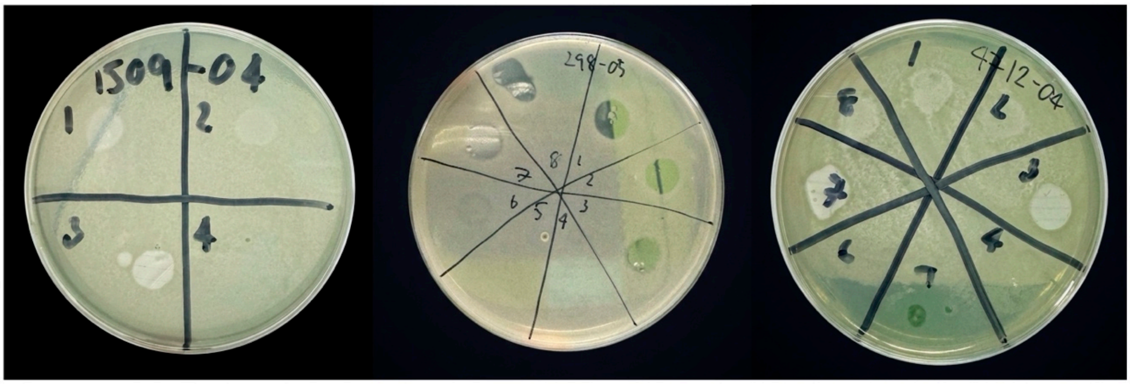

Plaque formation of phage vB_Pae_YuaWU01 is shown as indicated by label 1.
