# Supplementary material for: Isolation and Genomic Characterization of Lytic Caudoviricetes Bacteriophage vB_Pae_YuaWU01 Targeting Multidrug-Resistant Pseudomonas aeruginosa from Hospital Sewage in Southern Thailand
Source: Life (Basel). 2026 Apr 28;16(5):734. doi: 10.3390/life16050734 (PMC13208608; doi:10.3390/life16050734)
Supplement: Supplementary file 1 [file life-16-00734-s001.zip › Table S1 and S2 .pdf]

Table S1. Host range of phage vB_Pae_YuaWU01

	MDR Strains	Phage vB_Pae_YuaWU01 Lytic Activity
1	*P. aeruginosa* ATCC15692 (Host strain)	+
2	PA 541-10P	+
3	PA 5259-03	-
4	PA 1819-11N	-
5	PA 1450-11	+
6	PA 3207-10	-
7	PA 1869-10	-
8	PA 5106-10	-
9	PA 1088-04	-
10	PA 2659-04	-
11	PA 1189-10	-
12	PA 3194-04	-
13	PA 1985-10	-
14	PA 3212-04	-
15	PA 3259-04	-
16	PA 298-05	+
17	PA 774-04	-
18	PA 462-04	-
19	PA 4293-04	-
20	PA 4553I-04	-
21	PA 1509-04	+
22	PA 4712-04	+
23	PA 1918-10	-
24	PA 4222-03	-
25	PA 3685-10	-
26	PA 1181-10	+
27	PA 362-11	-
28	PA 1092-10	-
29	PA 1869-10	-
30	PA 5064-10	+
31	PA 3285-10	-
32	PA 207-10	-
33	PA 1819-11N	-
34	PA 3809-04	+
35	PA 4087-03	-

	MDR Strains	Phage vB_Pae_YuaWU01 Lytic Activity
36	PA 1695-10	-
37	PA 4795-03	-

Table S2. Comparison of phage gene annotations produced by Bakta and Pharokka

ORF	Start	End	Strand	Bakta	Pharokka
ORF1	3	1550	+	Terminase	terminase large subunit

ORF	Start	End	Strand	Bakta	Pharokka
ORF2	1608	3137	+	DUF4055 domain-containing protein	portal protein
ORF3	3134	3253	+	DUF2933 domain-containing protein	hypothetical protein
ORF4	3253	3840	+	GIY-YIG domain-containing protein	homing endonuclease
ORF5	3827	5035	+	Phage-Mu-F domain-containing protein	head morphogenesis
ORF6	5104	5886	+	Structural protein	head scaffolding protein
ORF7	6082	7014	+	Major capsid protein	major head protein
ORF8	7120	7692	+	Structural protein	virion structural protein
ORF9	7712	8317	+	DUF4140 domain-containing protein	hypothetical protein
ORF10	8345	8515	+	Structural protein	hypothetical protein
ORF11	8751	9017	+	Lipoprotein	Rz-like spanin
ORF12	9085	9420	+	Holin	holin
ORF13	9405	10130	+	Endolysin	endolysin
ORF14	10130	10648	+	DnaT-2 domain-containing protein	head-tail adaptor Ad1
ORF15	10652	11020	+	Structural protein	head closure Hc1
ORF16	11017	11469	+	Structural protein	tail terminator
ORF17	11503	13035	+	Major tail tube protein	minor tail protein
ORF18	13098	13556	+	Structural protein	tail assembly chaperone
ORF19	13580	13846	+	Tail chaperonin	tail chaperonin
ORF20	13830	14276	+	Virion structural protein	tail completion or Neck1 protein
ORF21	14279	17104	+	TMP-3 domain-containing protein	tail length tape measure protein
ORF22	17114	18634	+	Structural phage protein	structural protein
ORF23	18648	19640	+	Structural phage protein	structural protein
ORF24	19642	21312	+	Tail assembly structural protein	tail assembly protein
ORF25	21309	22118	+	Phage-BR0599 domain-containing protein	tail assembly protein
ORF26	22132	22365	+	Structural phage protein	tail assembly chaperone
ORF27	22365	22568	+	FluMu-N domain-containing protein	tail assembly chaperone
ORF28	22552	24723	+	Phage-tail-3 domain-containing protein	tail protein
ORF29	24723	25208	+	Structural protein	hypothetical protein
ORF30	25276	25620	+	DUF4164 domain-containing protein	hypothetical protein

ORF	Start	End	Strand	Bakta	Pharokka
ORF31	25617	25835	+	hypothetical protein	hypothetical protein
ORF32	26048	27742	+	Helicase C-terminal domain-containing protein	DNA helicase
ORF33	27750	28103	+	DUF126 domain-containing protein	hypothetical protein
ORF34	28163	28708	+	Mu-like prophage host-nuclease inhibitor protein Gam	hypothetical protein
ORF35	28850	29689	+	Single-stranded DNA-binding protein	hypothetical protein
ORF36	29791	30984	+	UvrD-C-2 domain-containing protein	Dda-like helicase
ORF37	31006	32868	+	adenosylcobalamin-dependent ribonucleoside-diphosphate reductase	ribonucleoside-diphosphate reductase large subunit
ORF38	32865	33053	+	Structural protein	hypothetical protein
ORF39	33013	33519	+	UDG domain-containing protein	hypothetical protein
ORF40	33619	34302	+	Helix-turn-helix domain-containing protein	hypothetical protein
ORF41	34313	34576	+	Transposase	hypothetical protein
ORF42	34586	35461	+	Glycinyltransferase	hypothetical protein
ORF43	35463	36299	+	Glycyl-dTMP PLP-dependent decarboxylase	threonine dehydratase
ORF44	36296	36676	+	Putative HNH endonuclease	endonuclease
ORF45	36738	38180	+	Radical SAM Nalpha-GlyT isomerase	DNA repair photolyase
ORF46	38235	38798	+	5-hmdU DNA kinase	terminase small subunit
ORF47	38810	39790	+	aGPT-Pplase1 domain-containing protein	hypothetical protein
ORF48	39802	39915	+	hypothetical protein	hypothetical protein
ORF49	39912	40967	+	Deoxyuridylate hydroxymethyltransferase	thymidylate synthase
ORF50	40980	41615	+	5'-deoxynucleotidase	hypothetical protein
ORF51	41590	41817	+	Phosphohydrolase	hypothetical protein
ORF52	41786	42172	+	DUF3310 domain-containing protein	1.7 protein
ORF53	42172	44166	+	DNA-directed DNA polymerase	DNA polymerase I
ORF54	44163	44477	+	Zn-ribbon containing protein	hypothetical protein

ORF	Start	End	Strand	Bakta	Pharokka
ORF55	44474	44935	+	CMP/dCMP-type deaminase domain-containing protein	dCMP deaminase
ORF56	44928	45326	+	DNA-LIGASE-A3 domain-containing protein	DNA helicase
ORF57	45311	45637	+	Repressor	transcriptional repressor
ORF58	45639	46142	+	GIY-YIG domain-containing protein	homing endonuclease
ORF59	46145	48556	+	DUF5906 domain-containing protein	DNA primase
ORF60	48567	48782	+	Type II toxin-antitoxin system HicA family toxin	hypothetical protein
ORF61	48894	49259	+	Holin	hypothetical protein
ORF62	49298	50476	+	HATPase-c-4 domain-containing protein	HATPase
ORF63	50473	50811	+	Transcriptional regulator	hypothetical protein
ORF64	50823	51092	+	Secreted protein	hypothetical protein
ORF65	51089	51700	+	Nucleotide pyrophosphohydrolase	MazG
ORF66	51697	52173	+	Mediator of RNA polymerase II transcription subunit 30	hypothetical protein
ORF67	52170	52499	+	Nitrogenase-stabilizing/protective protein NifW	hypothetical protein
ORF68	52518	52814	+	DLH domain-containing protein	hypothetical protein
ORF69	52817	53104	+	Structural protein	hypothetical protein
ORF70	53109	53378	+	Type II toxin-antitoxin system ParD family antitoxin	Portal
ORF71	53375	53542	+	Arm-DNA-bind-3 domain-containing protein	hypothetical protein
ORF72	53521	54138	+	Serine/threonine protein kinase	hypothetical protein
ORF73	54135	54416	+	Phage protein	hypothetical protein
ORF74	54418	54990	+	Rv2179c-like domain-containing protein	exonuclease
ORF75	54983	55174	+	nan	DksA-like zinc-finger protein
ORF76	55275	55883	+	Phage protein	hypothetical protein
ORF77	56016	56279	+	Virion structural protein	hypothetical protein
ORF78	56330	57214	+	DNA binding protein	hypothetical protein
ORF79	57218	57688	+	Diguanylate-cyclase GGDEF domain protein	Cyclic di-GMP metabolism protein%2C combines GGDEF

ORF	Start	End	Strand	Bakta	Pharokka
					and EAL domains with a 6TM membrane domain
ORF80	57783	58193	+	Antirestriction protein	anti-restriction protein
ORF81	58251	58586	+	Phage protein	hypothetical protein
ORF82	58601	58813	+	Pterin-binding domain-containing protein	hypothetical protein
ORF83	58810	59205	+	CCHC-type domain-containing protein	hypothetical protein
ORF84	59202	59390	+	NUDIX hydrolase	hypothetical protein
ORF85	59394	59648	+	Structural protein	hypothetical protein
ORF86	59645	60067	+	DUF1122 domain-containing protein	hypothetical protein
ORF87	60064	60270	+	ClpX-type ZB domain-containing protein	hypothetical protein
ORF88	60270	60635	+	Peptidase-S9 domain-containing protein	hypothetical protein
